# Droplet impact dynamics on stretched textiles

**DOI:** 10.1038/s41598-025-89855-8

**Published:** 2025-02-22

**Authors:** Nonu Varghese, Corinne A. Stone, Miguel A. Quetzeri-Santiago, J. Rafael Castrejón-Pita

**Affiliations:** 1 Department of Mechanical Engineering, University College London, Torrington Place, London, WC1E 7JE UK; 2 School of Engineering and Material Sciences, Queen Mary University of London, London, E1 4NS UK; 3 Defence Science and Technology Laboratory, Porton Down, Salisbury, SP4 0JQ UK; 4 Instituto de Investigaciones en Materiales, Universidad Nacional Autónoma de México, Cd. Universitaria, 04510 Mexico City, Mexico

**Keywords:** Fluid dynamics, Engineering

## Abstract

Preventing droplets from penetrating fabrics is critical in surgical wards and the battlefield, where biological and chemical hazards are dispersed and transmitted in droplet form. Here, we study the interaction between droplets and a nylon textile using high-speed imaging. We explore various droplet impact velocities and liquids to understand the influence of liquid characteristics on the impact behaviour. Importantly, we investigate the impact dynamics of droplets on textiles subjected to various tensile forces. Critical phenomena, such as droplet penetration and capture, are analyzed. We find that the critical impact velocity for droplet penetration increases as the stretching tension in the textile decreases. Furthermore, we present a simple model to predict the critical conditions for droplet penetration and capture that takes into account the surface tension, the droplet size, density and speed, the tensile load and the textile contact angle. We validate the model through experiments, demonstrating a strong agreement. These insights hold significant implications for the design of protective garments, such as face-masks and water-repellent clothing.

## Introduction

Rainfall, a ubiquitous natural occurrence, often compels us to seek cover to prevent us from wetting. Commonly, we turn to umbrellas or to other specialized clothing for shelter. Unfortunately, rainwater is not the only substance that comes in droplet form; viruses^1^, and other hazards^2^ are often transmitted by droplets too. For example, healthcare professionals have found it imperative to equip themselves with high-quality protective garments to shield against virus-laden liquid droplets^3^. Well-designed face masks have proven to be an effective defense against viruses such as SARS-CoV-2, responsible for COVID-19^4,5^. In the battlefield, uniforms are designed to prevent the penetration and contact of harmful biological and chemical liquid components to the skin^6,7^. These everyday experiences illustrate instances where droplets interact with textile-like materials to prevent us from becoming wet or coming into contact with liquids. In contrast, capturing droplets in textiles is sought in various industrial applications, such as in fog collectors where plastic meshes are exposed to winds to trap and harvest water^8^. In addition, the wicking and capture of droplets are desired in the printing and coating of fabrics to assure a good colour registry and graphic transfer. Indeed, the manufacturing of fabrics is shifting towards the adoption of inkjet methods for printing graphics, mainly because of its digital nature, accuracy and resolution^9^. Mesh-like textiles have been the chosen material for clothing for millennia, thanks to their breathable properties and ease of manufacturing. It is clear that a good understanding of droplet dynamics on and within fabrics is crucial to the development of the new generation of clothing.

The study of droplet impact dynamics has its roots in the work by A.M. Worthington’s in 1895^10^. Since then, a significant number of past works have studied the impact of droplets on flat and non-permeable surfaces. It has been found that various factors influence these dynamics, e.g. the liquid and surface characteristics, and the ambient gas^11,12^. In brief, an impacting droplet can, among other outcomes: i) spread and recede to an equilibrium diameter (this process is called smooth deposition), ii) spread, recede and bounce off the surface, iii) spread and fragment during receding (receding breakup), or iv) splash soon after impact^11,13,14^. The interaction between the surface and the liquid is often characterized by their wettability, through the contact angle^15^. Droplet impact and subsequent behaviour are often parametrised by dimensionless numbers, with the Reynolds, $Re = \frac{\rho D U}{2\mu }$, and Weber numbers, $We = \frac{\rho D U^2}{2\sigma }$, being the most common^16,17^. Here, $\rho$ is the fluid’s density, $\mu$ its viscosity, $\sigma$ is the surface tension, *D* denotes the droplet diameter prior impact, and *U* is the impacting speed. The substrate mechanical characteristics are also known to affect droplet dynamics, e.g. droplets impacting a soft solid visibly behave different than when impacting a solid surface. Droplets impacting deformable solids change their shape during spreading due to the surface absorbing part of the energy that would otherwise contribute to fragmentation and splashing^18^. In fact, splashing on a soft substrate is promoted by the substrate’s stiffness; it is easier to splash on hard solids than on soft deformable substrates^18^. Other works, have demonstrated that droplets impacting a hydrophobic *flexible* substrate induce a simple harmonic oscillation that shortens the contact time between the liquid and the substrate, when compared to a rigid substrate^19^. Other works have determined that the droplet impact behaviour on stretched membranes is dependent on the tensile strength applied to the membrane^20^.

**Figure 1:**
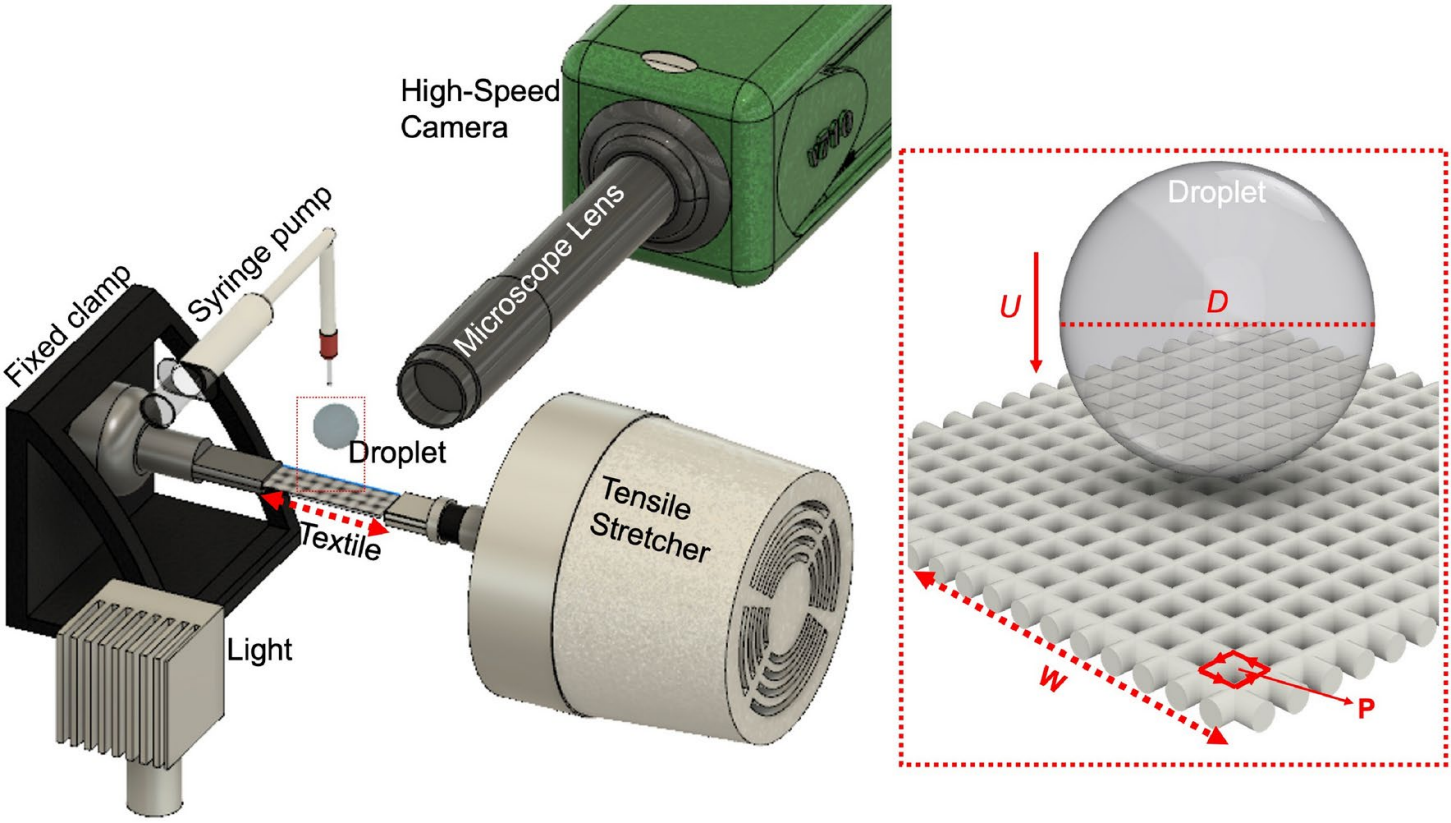
Schematic view of the experimental setup used for the visualisation droplet impact on the textile samples. The setup integrates a syringe pump, a high-speed camera (Phantom V710), an LED light source, and an uni-axial Electroforce tensile stretcher. On the right-hand side inset, red box, key parameters such as the impact velocity (*U*), droplet diameter (*D*), the mesh opening perimeter (*P*), and the applied load direction (*W*) are illustrated.

Past studies have also examined droplets impacting on porous and permeable substrates. In 2003, Lorenceau and Quéré studied droplets impacting on a single hole punctured on a solid surface finding three different behaviours: a) at high speeds, the droplet passes through the hole, b) at low speeds, the droplet spreads across the surface, and c) at other intermediate speeds, the droplet initially passes through the hole but is subsequently pushed back towards the substrate. This last mechanism is referred to as the *capture* of the droplet^21^. A common occurrence during capture is that upon impact, in addition to the droplet permeating through the hole, a lamella spreads on the upper surface of the substrate.

Droplet impact on textile permeable substrates is an area gaining traction due to its relevance in forensics and fabric manufacturing. In 2018, Zhang et al. demonstrated that droplet penetration and capture behaviour is determined by the textile’s porosity and speed of impact^6^. In fact, these studies also indicated that the dynamics are unaffected by the textile wettability^6^. A year later, in 2019, Kooij et al. studied the spray formation following droplet impacting on a mesh finding that the impacting velocity, the wetting behavior, and the mesh rigidity affect the spraying behaviour^22^. In 2021, de Goede et al. found that, during droplet spreading on fabrics, viscous losses hinder the maximum spreading diameter^23^. Furthermore, it has been found that complete penetration can be suppressed by a droplet standing on the textile prior the impact^24^. In the context of COVID-19 and other disease transmission, the recent works of Krishan et al. and Sharma et al., have shown that single layered textile face-masks contribute to drop atomisation and that a minimum 3 layered cotton masks are required to avoid droplet penetration^5,25,26^. In works in 2023, Abouelsoud et al., studied the impact of silicon oils on oleophilic meshes finding that the threshold for liquid penetration (where droplets breakup/separate from liquid captured within the mesh) is given by $Re = 7.5 We / (We -8)$, with less droplets penetrating at higher viscosities^27^. In addition, Zong et al. determined that the dynamics resulting from a droplet impact on a mesh are independent on the wettability of the mesh material^28^.

In forensic sciences, the dynamics of droplet impact on fabrics, and other substrates, are crucial^29^. In 2007, Knock and Davison developed an empirical relationship to determine the direction and speed of a blood droplet based on the aspect ratio of the bloodstain, or footprint^30^. As part of other works, Bloodstain Pattern Analysis (BPA) on cotton textiles has been used to determine the role of the impacting velocity and droplet properties on fabrics wicking dynamics^31^. In 2013, C.D. Adam, by using painted surfaces of different roughness, concluded that the spreading behaviour of impacting blood droplets is controlled by the substrate characteristics^32^. In all these past works, textile targets have either been mounted on solid surfaces^31^ or kept stretched at a constant tensile force^6^; the role of the fabric’s tensile strength on the droplet dynamics had yet to be explored.

Textile materials are inherently flexible and porous; when a bounded textile is impacted by an object, it undergoes to a shape change, oscillations, and eventually comes to a rest. While liquids possess the ability to penetrate through the material, during this process, some of the droplet impacting energy is dissipated within the textile. Depending on the textile stretching strength, the resulting oscillations might be visible, unless the textile is sufficiently stretched to the point where its deformation is negligible^6,23^. In 2019, Cheng et al. conducted experiments, and volume-of-fluid simulations, to analyze droplet impact on nylon textiles under various tensile strengths, these ranging from 0 to 400 N/m. Their results indicated a minor dependency of both the peak impact force and the vibration frequency and the tensile force^33^. In addition, they found that the maximum spreading diameter remains unchanged by the stretching. In contrast, on the same year, Kooij et al. qualitatively varied the mesh tension to observe that a loose mesh results in less droplet volume penetrating the textile; they also identified a critical tensile force above which no penetration occurred^22^. In daily life, textiles are commonly found stretched, loose, or both. The fabric of an umbrella is kept evenly stretched, and in shape, by its rib supporting structure to form a protective canopy. During printing, fabrics are kept stretched to achieve a smooth and wrinkle-free surface where graphics can be transferred at a high resolution. In contrast, wearable textiles can be found loose, or tensioned, according to its location on the body or use^34^. In all these scenarios, textiles can interact with liquid droplets. In this paper, we explore the dynamics of droplets impacting on textile surfaces subject to various controlled tensile forces *W*. We experimentally studied the droplet impact dynamics on nylon textiles at various applied loads. Additionally, we explore the effect of wettability on the penetration dynamics by using silicon oil and water droplets. We also present a simple model that takes into account surface tension and wettability effects to parametrise the penetration/no-penetration conditions; the model having a good agreement with our experiments.

**Table 1:** Droplet Liquid Properties at laboratory conditions, i.e. 25 Celsius. Contact angle measured on a flat solid nylon sample.

Liquid	$\mu$	$\sigma$	$\rho$	*D*	$\theta$
	mPa s	mN/m	$\hbox {kg/m}^3$	mm	degrees
Water	0.89	72.8	997	2.92	51
Silicon Oil	1.75	18.7	876	2.28	$\lesssim 10$

**Figure 2:**
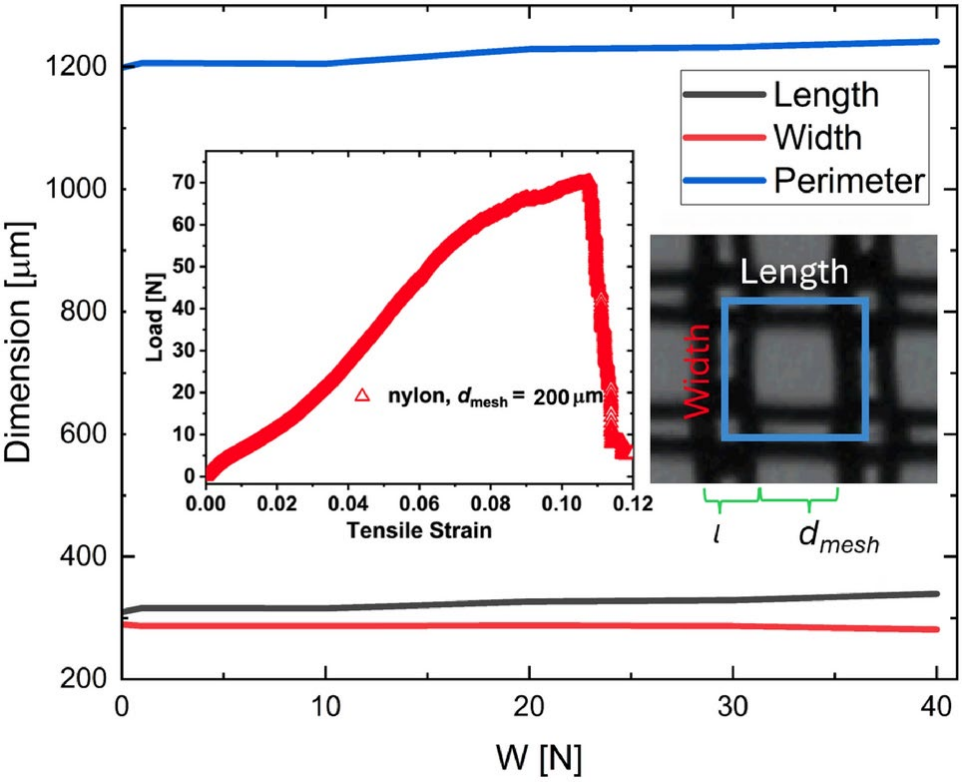
Textile opening perimeter in terms of the applied stretching load. As observed, there is minimal deformation, i.e. less than $< 5\%$ at all loads. The inset shows the results of a standard destructive tensile test applied to the textile. As observed, the nylon textile can withstand a maximum force of $\approx$ 72 N before snapping. As consequence, and to operate within a range away from textile breaking, our experiments were restricted to the 0 to 40 N range. The optical microscope image shows the nylon textile and its characteristic sizes.

## Experimental method

The experimental setup is illustrated in Fig. 1. Individual droplets were generated using a syringe pump through a blunt-end stainless steel needles; two inner diameters were used: 0.83mm and 0.41mm (22 and 18 gauge, from Metcal). In this work, we have used distilled water ($\mu =$ 0.89 mPa s) and silicone oil ($\mu =$ 1.75 mPa s, Sigma-Aldrich); other liquid properties are given in Table 1. The flow rate of the syringe pump was kept constant, at 1.5$\hbox {cm}^3\hbox {h}^-1$, at this rate droplets acquired a spherical shape at the point of impact. The droplets were let to fall free to impact the textile sample; the speed of the droplet was controlled by adjusting the fall height from the needle to the substrate. The textile was cut into rectangular samples of the same size. Samples were secured from their opposite ends to a ± 200N load cell which in turn was attached to an Electroforce Uniaxial Tensile Stretcher; the stretching system has minimum stretching load steps of 1.0 N. The stretcher unit was run with one linear motor and a fixed clamp, as seen in Fig. 1, both mounted on a rigid optical table. The textile was clamped between the load cell and the fixed clamp by a 3D printed (in-house designed) jaw structure with hand-tightening screws. Between the jaws, the textile was bound straight and wrinkle-free. The nylon textiles are commercially available nylon meshes (from Cadish Precision Meshes^35^), composed of single nylon fibres (monofilament) arranged perpendicular to each other, creating square-shaped openings. In this work, the nylon textiles had a measured mesh size/opening of $d_{mesh}= 200$ $\mu$m, or a opening perimeter of $P = 800$ $\mu$m, and a thread diameter of $l =$ 105 $\mu$m, as measured from the fibre centers (Fig. 1).

As a reference, equilibrium contact angles were measured using sessile droplets on a solid smooth nylon block. Water droplets on nylon have a equilibrium contact angle of 51 $\pm 3$ degrees and silicon oil on nylon have a contact angle of $\lesssim 10$ degrees; in our calculations below we used a value of 10 degrees. Sessile water droplets on the nylon meshes remain entirely on the top surface, but silicon oil droplets wet, traverse through the mesh, and remain suspended beneath.

Tensile breaking tests were performed to determine the maximum stretching load for the nylon textile before failing. The destructive tests utilized an Instron 5967 machine equipped with a 1 kN tensile stretching unit, operating at a stretching speed of 10 mm/min. Textile samples broke reliably at $\approx$70 N as seen in Fig. 2. Thus, to avoid any damage to the textiles during experiments, our experiments were limited to the range from $W = 0$ to 40 N. As seen in further sections, within this range we captured a clear asymptotic behaviour at tensile forces > 10 N; higher loads were not required.

All our textile samples were cut to a dimension of $10 \times 80$ $\hbox {mm}^2$. The impacting events were captured using a Phantom V710 high-speed camera, equipped with a 12$\times$ Navitar lens and a $2\times$ extension tube. Recording speed was set to 13,000 frames per second with an exposure time of 5.0 $\upmu$s. Shadowgraph illumination was used to visualise the impact, where the droplets were illuminated from the back by either a 500 or a 100 W LED lamp. The effective resolution for the images was between 78 to 110 pixels per millimeter. Images were analyzed using MATLAB to extract the impacting velocity and radius of droplets.

**Figure 3:**
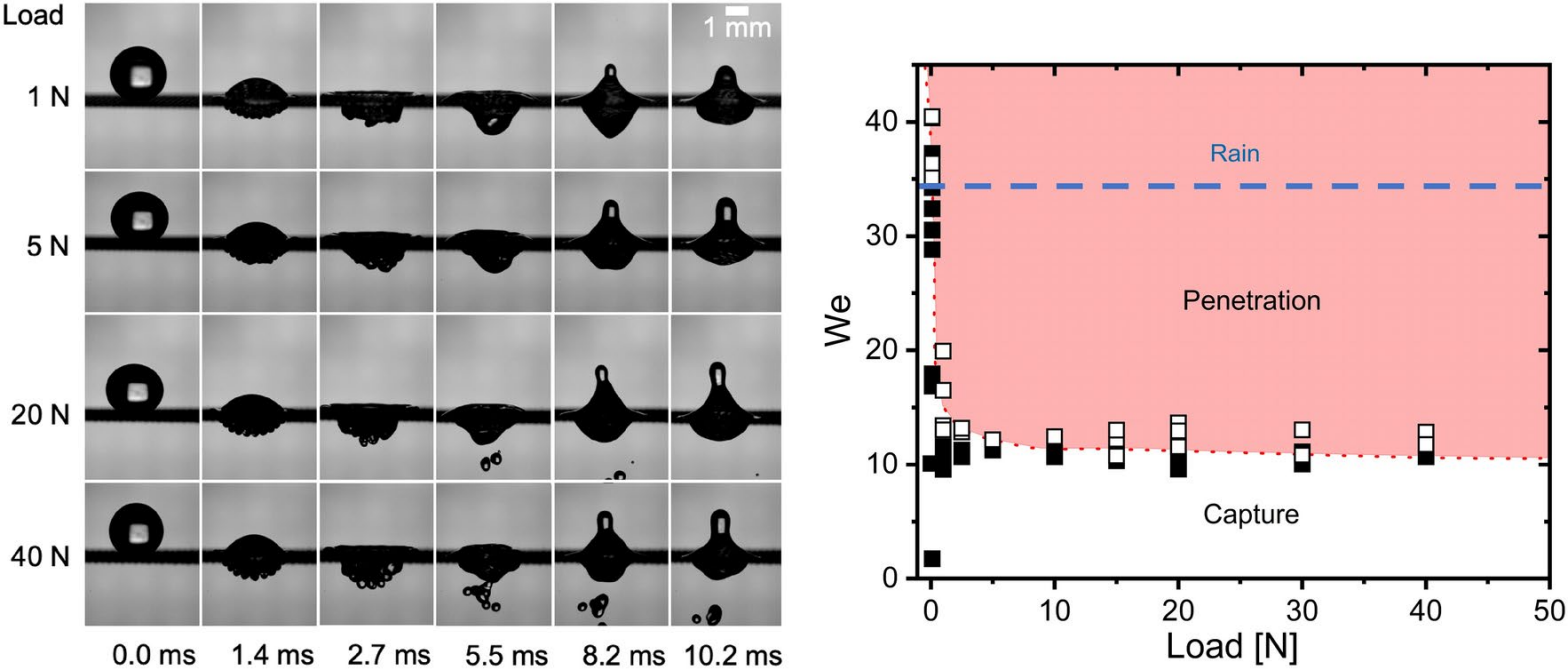
Left: Snapshots of water droplets impacting a, $d_{mesh}= 200$ $\upmu$m, nylon textile under different tensile loads. In all cases, *We* = 11.55±0.17, *U* = 0.76±0.01 m/s and *D* = 2.86±0.06 mm. The applied tensile forcing ranges from $W =$ 1.0 to 40.0 N. As observed, within our conditions, the droplet impacting behavior transitions from capture (*W* = 1.0 to 5.0 N) to penetration (*W* = 20 and 40 N). Right: The Weber number in terms of the applied stretching load; the red-shaded region highlights the conditions for droplet penetration. The dashed line shows the Weber impacting conditions for raindroplets. As seen, rain can only be captured by the nylon textile when loose.

## Results and discussion

As discussed in the introduction, past works have determined that the droplet impact and penetration behaviour depend on the mesh (opening) size and the impact speed^6^. To account for any pore size variations, due to the tensile stress, conventional digital microscopy was used to visualise, and measure, the mesh opening size as the stretcher varied the load. Figure 2 presents the mesh variations of: the opening perimeter *P*, width and length, in terms of the applied load to the textile. As seen, only small perimeter changes are observed, with *P* changing less than 4 $\%$ over the full range. Our results also show that most of the textile variations occur on the direction parallel to the applied stretching, i.e. the mesh length (as seen in the plot of Fig. 2). As seen, in the direction of the applied stretching, the mesh length changes less than 10%. According to the results by Zhang et al., these size variations should be negligible to the droplet penetration dynamics^6^. Therefore, in our experiments, we can safely assume that changes in the mesh size due to stretching should not affect the penetration dynamics as the load is varied. The inset of Fig. 2 shows the relationship between stress (force applied per unit area) and applied load as a nylon textiles undergo the destructive testing. During these experiments, a constant loading rate of 10.0 mm/min was applied until material failure.

### Penetration behaviour

**Figure 4:**
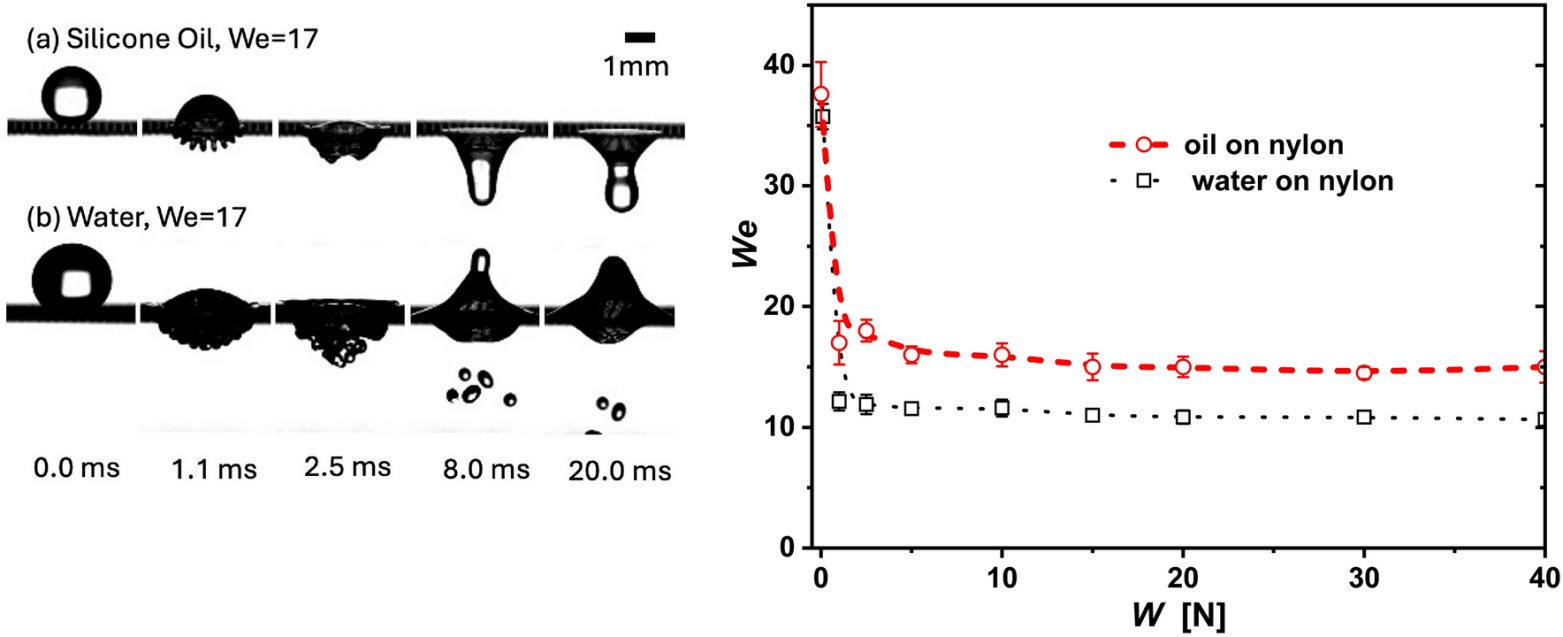
Here, we show the high-speed impact of both water and silicon oil droplets on a nylon mesh stretched at a tensile forcing of $W = 2.5$ N. The top two rows depict droplets impacting the mesh, both at *We* = 17 number, with oil being captured but water resulting in forced penetration. The bottom row exemplifies a case of forced penetration, found at a high Weber numbers, at this conditions both liquid penetrating vigorously. The graph to the right shows the threshold of penetration for both water and silicone oil in terms of the Weber number and the tensile forcing; a clear offset is visible.

At a given tensile force, droplets impacting a textile can result in one of four possible distinct outcomes. In the first scenario, at low speeds, the droplet uniformly spreads across the upper surface of the textile; this behaviour seen in all the cases included in the complementary video. In the second case, at moderate impacting velocities, the droplet (partially or fully) penetrates the textile but firmly adheres to the underside, a phenomenon referred to as “capture”, this behaviour is exemplified at W = 1 and 5 N loads in Fig. 3 (left). The third possible case arises at high speeds when the droplet traverses the fabric and disintegrates into one, or a few, smaller secondary droplets. This typically occurs near the critical penetration speed, where the droplet initially breaches out, passes the textile, and then fragments into secondary droplets. Finally, at very high speeds, the fourth outcome is marked by a forceful penetration, resulting in the violent fragmentation of the droplet into numerous secondary droplets; Fig. 4 (left) exemplifies this behaviour. Our experiments have shown that these different dynamics can also be found, at a constant speed, by varying the stretching force on the textile samples, as seen in Fig. 3 (left). In a previous study, Zhang et al. (2018) found that the Weber number effectively separates the penetration-capture transitional behaviour of low-viscosity droplets impacting textiles^6^. In a subsequent study, Abouelsoud et al. (2023) found that viscosity only becomes relevant to droplet penetration^27^ at $\mu > 10$ mPa s. In our experiments, the viscosity is low, which results in the Weber number, which quantifies the relative importance of inertia against surface tension, effectively separating the penetration-capture transitional behaviour as seen in Fig. 3(right).

Figure 3(right) summarises the penetration behavior in terms of the tensile forcing for water droplets impacting the nylon mesh. Notably, these results demonstrate that, as the applied load increases, droplet penetration is promoted until an asymptotic threshold value is achieved. After this asymptotic value is reached, increasing the stretching load does not affect the penetration behaviour. As seen for water on nylon, Fig. 3(left), at low tensile stretching forces, the full volume of the impacting droplets are retained, or captured, within the textile surface. Moreover, the impact, induces visible oscillations on the textile, as seen in the complementary video. In contrast, in the 20 N to 40 N load range, part of the liquid fully penetrates through the textile producing free satellite droplets at the other textile side, and the oscillation on the textile subside. In this region the textile, behaves approximately as a rigid material, as shown by the absence of textile oscillation in the supplementary video for stretching loads of W = 10 and 30 N. According to Villermaux and Bossa$^{37}$ (2009), raindrops have a typical size of $D=$ 1.6 mm and travel at a speed of $U=$ 1.77 $\hbox {ms}^{-1}$; under these conditions, raindrops achieve a $We \sim$ 35 at impact. As seen in Fig. 3, raindrops could penetrate the textile at all stretching conditions except for when there is no load; the critical penetration threshold for loose textiles is We = $36 \pm 2$.. At the lowest stretching force (no stretching), the capture-penetration threshold is found in the range from $We = 35$ to 37, with the threshold rapidly falling with increasing stretching. At a stretching load of $W =$ 1.0 N, a droplet with We = 11 penetrates the textile; this corresponds to only half the speed than at no stretching, i.e. from $U =$ 1.36 m/s at $W =$ 0.0 N, to $U =$ 0.74 m/s at $W =$ 1.0 N.

Further observations indicate that the liquid properties also affect the penetration behaviour; Fig. 4 illustrates the contrasting behavior between water and silicone oil droplets. Experiments revealed that water penetrates the textile at a lower Weber number than for silicone oil. Figure 4 highlight this difference: with the mesh stretched at $W = 2.5$ N, at an equal *We* = $17 \pm 1$, water penetrates but silicone oil is captured. Additionally, this behavior is reflected as a shift in the critical Weber number for penetration, across all tensile forces as seen in the Fig. 4 graph. We note that the overall behaviour, i.e. increasing the forcing decreases the speed required to penetrate, remains consistent for both liquids and that a clear offset on the results is evident. From Fig. 4, we conclude that the Weber number (*We*) alone cannot fully parameterise the influence of both surface tension and wettability on the liquid penetration threshold. Here, we expect the viscosity to play a negligible role as the Ohnesorge number $Oh = \sqrt{We}/Re<< 1$^36,37^. Our data supports the hypothesis that on a textile structure, inertia needs to overcome the strain energy associated with the textile deformation; when inertia is not dissipated as textile oscillations, droplet penetration is promoted^37,38^. Such conditions are found, for example, at high impact velocities, or at low impact velocities but on a highly tensioned textile. In the following section, we introduce a momentum balance argument to predict this penetration behaviour.

In this study, we have focused on Newtonian fluids, but other liquids may exhibit a complex rheology. In fact, the viscosity of many biologically (and commercially) relevant fluids vary with the shear rate. Indeed, the impact of biological-relevant liquid droplets, such as blood and saliva, into fabrics is of interest to safety, medical and hospitality clothing and uniform manufacturers. Under our conditions, the shear rates ($\gamma$) experienced during textile penetration can readily be estimated through the mesh size ($d_{mesh}=$ 200 $\mu$m) and the speed of impact ($U \approx 2.0$ m/s), i.e. $\gamma = U/d_{mesh} \sim 10^4$ $\hbox {s}^{-1}$. As evidenced by Laan et al. (2014) and Cherry and Eaton (2013), these shear rates remain inaccessible by current rheometers^39,40^. Consequently, performing well-characterised parametric experiments with complex fluids, at these shear rate scales, remains a task for the future. Despite these limitations, in recent years specific studies have looked at blood droplet impact, spreading and stains due to its relevance to forensic sciences^23,39^. Laan et al. (2014) demonstrated that blood shear-thins at low shear rate scales, while its behavior becomes Newtonian at high rates reaching a viscosity of $\mu \sim 4.8$ mPa s for $\gamma > 10^3$ $\hbox {s}^{-1}$. Their study also show that, during impact and spreading, the shear-thinning behavior of blood is irrelevant, with spreading only influenced by the high-shear rate viscosity. In addition, Gittings et al. (2015) observed that saliva also shear-thins^41^ to $\mu \sim 2.0$ mPa s for $\gamma > 10^3$ $\hbox {s}^{-1}$. Another interesting study on droplet impact on meshes is that by Abouelsoud et al. (2023), which investigated the effect of droplet viscosity on mesh penetration^27^. The study found that viscosity becomes relevant only at $\mu > 10$ mPa s. In another relevant study, Mehrizi et al. (2022) found that viscoelastic effects do not affect the penetration threshold of droplets impacting on meshes^42^. Based on this evidence, we expect the dynamics of blood droplets, saliva, and possibly other shear-thinning liquids, to be indistinguishable from that of low viscosity Newtonian fluids. Nevertheless, the study of the droplet penetration dynamics in terms of high shear rate rheology stands as an significant project for the future

**Figure 5:**
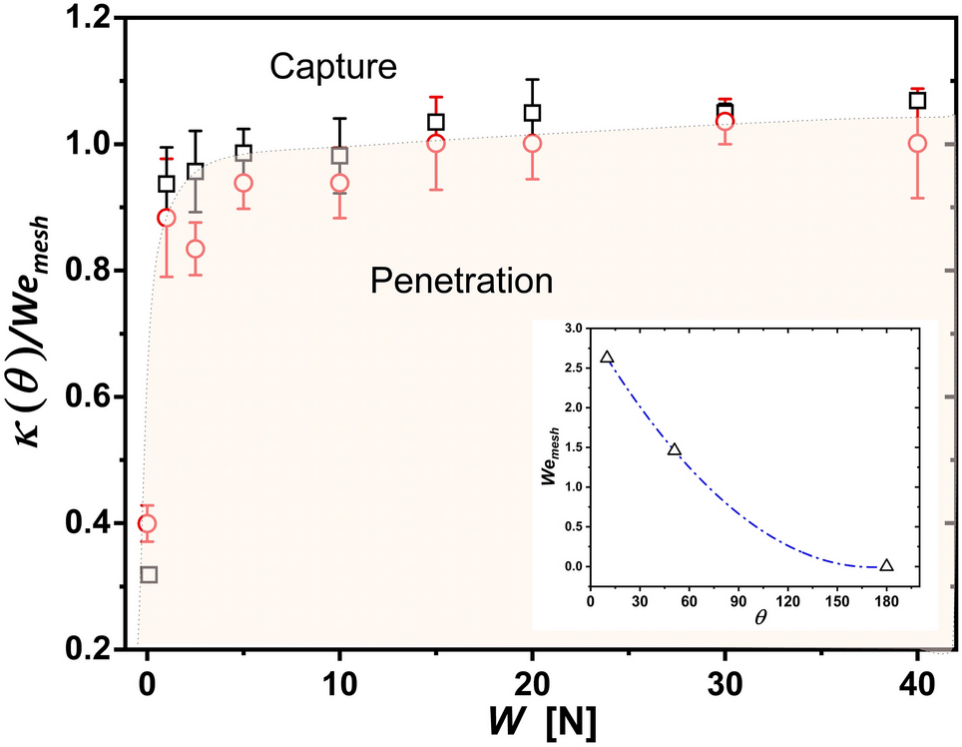
Critical conditions for the penetration-capture regime of droplets impacting a nylon textile in terms of the Weber number, the contact angle and the stretching loading. The shadowed area, penetration regime, is a guide for the eye.

### Penetration threshold

Zhang at al. found that the penetration threshold of $\mu =$10 mPa s droplets on differently coated nylon textiles, at low tensile strengths (*W* = 2.5 N), is solely determined by the Weber number and the mesh size^6^. Additionally, since $Oh = \sqrt{We}/Re<< 1$, for both silicon oil and water, we expect viscosity to not play a major role on the penetration dynamics^36,37^. As seen in Table 1, we face significant wettability differences between water and silicon oil on nylon. In fact, on the mesh, we measured the contact angle of silicon oil to be 27 degrees, while the contact angle of water was 90 degrees. Differences in penetration thresholds have been observed in hydrophobic meshes where the penetration threshold decreases for increasing advancing contact angles^36^. Furthermore, it has been found that hydrophilic fiber arrays dissipate more energy and prevent the penetration of water in furry mammals due to a prolonged spreading^43^. Consequently, evidence indicates that wettability plays a role on the penetration dynamics, with high contact angles promoting penetration.

Figure 4 (right) also indicates that the tensile strength has a dramatic effect on the penetration behaviour; loose textiles are harder to penetrate, with the thresholds rapidly achieving an asymptotic value as the stretching forcing is increased. This finding is in agreement with the work by Pepper et al. (2008)^20^, where flexible membranes were found to dissipate the droplet impacting kinetic energy through the substrate’s deformation. Pepper et al. found this dissipation to be inversely proportional to the tensile strength (*W*), i.e. $E_{deformation} \propto 1/W$. Taking into account these factors, the balance of fluid inertia, capillary forces, and substrate deformation, is given by:

1 $$\begin{aligned} \rho U^2 \propto \kappa (\theta )\frac{\sigma }{d_{mesh}}+ \zeta \frac{\rho ^2 d_{mesh}^2 U^4 }{W}, \end{aligned}$$

where $\kappa (\theta )$ is a dimensionless scaling factor that quantifies the influence of the textile’s wettability on the capillary forces. Similarly, $\zeta$ is a dimensionless parameter that accounts for the material characteristics of the textile, particularly its deformation response under impact. The rightmost term of Equation 1 is taken from Pepper et al. (2008)^20^ under the assumptions that the mesh size is the appropriate characteristic length scale, and that penetration occurs at the inertial time, when calculated using the textile thickness. Equation (1) can be written in terms of scaling arguments as

2 $$\begin{aligned} \zeta \frac{d_{mesh} \sigma }{W} + \kappa (\theta ) \frac{1}{We^2_{mesh}} \approx \frac{1}{We_{mesh}}, \end{aligned}$$

where $We_{mesh} = \rho d_{mesh} U^2 / \sigma$. As seen in Fig. 4, the asymptotic critical speeds for droplet penetration is achieved, for both liquids, at a tensile forcing $W > 5$ N. Consequently, at a larger stretching we can safely assume the momentum dissipation from textile deformation is minimal or negligible. Under this assumption, as $W \rightarrow \infty$, Equation 2 takes the form,

3 $$\begin{aligned} 1 \sim \kappa (\theta ) \frac{1}{We_{mesh}}. \end{aligned}$$

In addition to the condition at high tensile strengths, i.e. Eq. (3), we have one boundary condition for fully superhydrophobic meshes, i.e. $\theta = 180$ degrees, where the capillarity term becomes negligible as there is no interaction between the droplet and the mesh. Under these conditions, a power relationship arises between the contact angle and the Weber number, i.e. $\kappa (\theta ) = 1\times 10^{-4} \theta ^2 -0.035 \theta + 2.96$ (this is shown at the inset of Fig. 5). As seen in Fig. 5, this parametrisation now effectively separates the capture-penetration regimes for both liquids at all the stretching domain.

## Conclusions

In this work, we have investigated the impact behavior of silicone oil and water droplets on a nylon textile subject to various constant stretching loads. In our experiments, we have identified conditions under which droplets are either captured by the textile, or penetrate it, in terms of the impacting speed and stretching forces for two liquids. Our findings demonstrate that increasing the stretching load facilitates droplet penetration. Regarding the impact speed, our results align well with previous studies, confirming that higher speeds promote penetration, while lower speeds favor capture. By using two liquids of very different surface tension, we have explored the role of wettability on the penetration dynamics. As noted above, given the high shear rates experienced during penetration, we expect blood, saliva, and other shear-thinning droplets to behave like low-viscosity Newtonian fluids. However, studying textile penetration of complex droplets remains a significant area for future research.

By using different liquids, we have demonstrated that wettability influences the penetration threshold, with the Weber number alone unable to parameterise the behaviour. Our results demonstrate that wettability suppresses penetration, i.e. textiles with a lower contact angles are harder to penetrate. We have adapted a simple momentum balance model to account for wettability and capillarity dissipation with our experimental results showing a good agreement when dividing the behaviour between droplet capture or penetration. Our findings can provide valuable strategies for preventing liquid penetration in practical applications such as rain-wear, medical and military protective clothing, including facemasks. Implementing these findings can help reduce the transmission of diseases through droplets. In fog harvesting, slacked meshes would capture more water than when under tension. Loose textiles would promote the capture of droplets, while stretched textiles favour penetration.

Previous studies have demonstrated that material properties, such as the Young’s modulus, influence the outcome of droplet impacting on soft-solids^18^, including suppressing splashing. Future research could investigate the role of the textile stiffness and other material properties in the droplet penetration dynamics. In this work we have focused on simple, single yarn, textiles, but future works can expand into exploring different weaving patterns, or on textiles containing more than type of yarns. Another interesting direction is to study the dynamics of time-varying stretching loads, including textiles under vibration.

## Data Availability

The datasets used during the current study are available from the corresponding author on reasonable request.
